# A Strategy for Problem Solving of Filling Imbalance in Geometrically Balanced Injection Molds

**DOI:** 10.3390/polym12040805

**Published:** 2020-04-03

**Authors:** Krzysztof Wilczyński, Przemysław Narowski

**Affiliations:** Polymer Processing Department, Faculty of Production Engineering, Warsaw University of Technology, Narbutta 85, 02-524 Warsaw, Poland; p.narowski@wip.pw.edu.pl

**Keywords:** injection molding, filling imbalance, optimization, simulation

## Abstract

Simulation and experimental studies were performed on filling imbalance in geometrically balanced injection molds. An original strategy for problem solving was developed to optimize the imbalance phenomenon. The phenomenon was studied both by simulation and experimentation using several different runner systems at various thermo-rheological material parameters and process operating conditions. Three optimization procedures were applied, Response Surface Methodology (RSM), Taguchi method, and Artificial Neural Networks (ANN). Operating process parameters: the injection rate, melt temperature, and mold temperature, as well as the geometry of the runner system were optimized. The imbalance of mold filling as well as the process parameters: the injection pressure, injection time, and molding temperature were optimization criteria. It was concluded that all the optimization procedures improved filling imbalance. However, the Artificial Neural Networks approach seems to be the most efficient optimization procedure, and the Brain Construction Algorithm (BSM) is proposed for problem solving of the imbalance phenomenon.

## 1. Introduction

**Figure 1:**
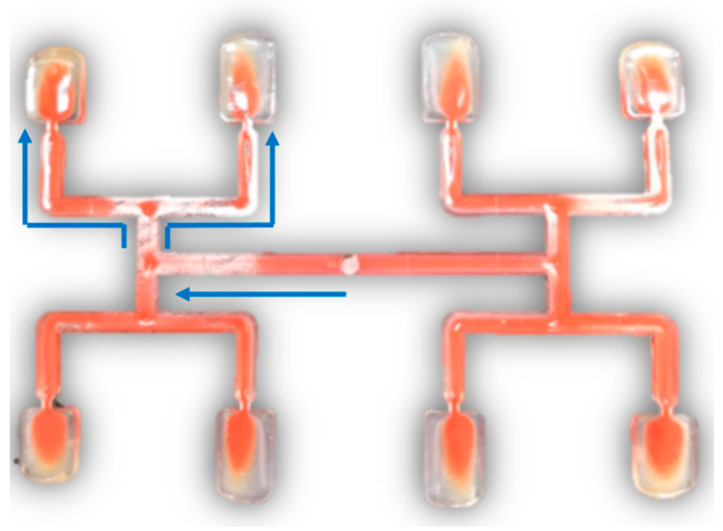
Filling imbalance in geometrically balanced injection molds.

The imbalance of polymer flow in geometrically balanced multi-cavity injection molds is a serious and difficult to handle problem in the injection molding process. This phenomenon may be observed in H-type runner layouts, which are depicted in Figure 1. At present, it is established that the imbalance results from the polymer melt flow shear gradients developed in the runner system, which in turn lead to non-symmetrical temperature and viscosity distributions. This is influenced and complicated by the runner’s geometry, thermo-rheological material characteristics, and injection molding process parameters [1,2,3,4,5,6,7].

The imbalance phenomenon has been investigated extensively for years by scientists and engineers [1,2,3,4,5,6,7,8,9,10,11,12,13,14,15,16,17,18,19,20,21,22,23,24,25]. However, there is not any commonly accepted procedure for problem solving. A detailed discussion of the literature review has been recently presented by the authors of this paper [1,2]. This review is summarized below.

The fundamental research was carried out by Beaumont and his co-workers [3,4,5,6] as well as by Reifschneider [7] who tried to explain the phenomenon of filling imbalance. Beaumont et al. developed the commercial technique called melt rotation technology to diminish the filling imbalance [3,4,5,6]. In addition to the basic design with one correction element, double corrections and a circular element were proposed. However, these designs were not studied by simulation or experimentation. Another approach has been proposed by Huang [8], who applied profiled channels instead of straight channels. 

Petzold [9] and Fernandes [10] developed the concepts of optimizing the temperature field of the flowing polymer. Rhee [11,12] proposed flow balancing by using the throttle valves in the runners which were activated when the mold temperature rises. Schwenk [13] studied the effect of mold venting on flow balancing.

Recently, the filling imbalance has been investigated with advanced techniques. Gim et al. [14] used a mold equipped with thermocouples and in-direct pressure sensors, and they observed that the filling imbalance may be inverse. Li et al. [15] studied the phenomenon by direct in-mold observations using a mold with a sight glass. 

The mold filling simulation software [16,17,18,19] was not able to diagnose the shear effect on the polymer flow for a long time. The use of simple 1D beam elements, and 2D or 2.5D approach did not allow to predict the shear phenomena properly. Thus, new simulation concepts have been proposed to study the problem [20,21]. These included three-dimensional non-isothermal non-Newtonian flow and inertia effects. Convection was predicted using 3D velocity vector, and this allowed the simulation of the temperature field around the runners properly. Moreover, a flexible meshing was applied to provide high-resolution mesh for the runners and the cavity. Several simulation studies have confirmed this approach [22,23,24,25]. 

Summarizing, so far, the filling imbalance has been studied experimentally and to a less extent by simulation for geometrically balanced systems and for basic melt rotation solutions only.

**Figure 2:**
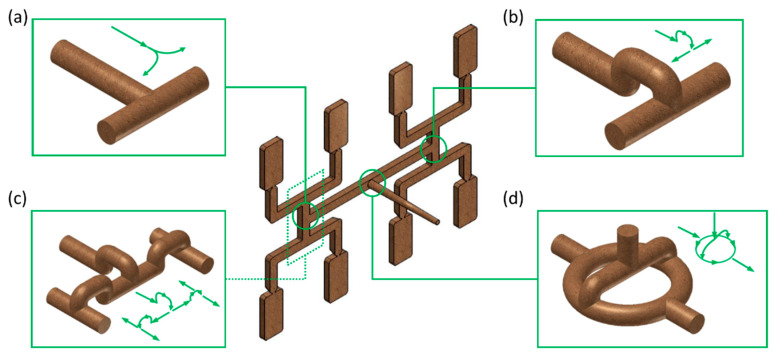
Geometry of runner layouts: (**a**) standard *GS*, (**b**) one correction element *G*1, (**c**) two correction elements *G*2, and (**d**) circled element *G*3 [2].

Recently, extensive experimental and simulation studies on the filling imbalance have been performed by the authors of this paper [1,2]. Balancing the polymer flow between cavities has been investigated at various operating conditions using various runner systems, which are depicted in Figure 2. The experiments indicated that the process parameters: injection rate, mold and melt temperature as well as the runners’ layout geometry significantly affect the filling imbalance. However, the imbalance has never been eliminated completely. A special simulation technique was developed to simulate the phenomenon properly, including inertia effects and 3D tetrahedron meshing of at least 12 layers as well as meshing of the nozzle. It has been concluded that the thermo-rheological characteristics of the material, defined by the parameters of the Cross-WLF model, i.e., the index flow, critical shear stress (relaxation time), and zero shear viscosity, as well as characterized by the thermal diffusivity and heat transfer coefficient, significantly affect the filling imbalance. This is strongly dependent on the runners’ layout geometry and process operating conditions: flow rate and shear rate. The main conclusion of these studies was that there is no universal procedure to overcome the filling imbalance, and the only reasonable approach is to optimize the process parameters (material parameters, operating conditions, as well as runners’ geometry and layout) for the currently implemented process. 

In this paper, a novel optimization approach has been presented to solve the filling imbalance problem. Three optimization procedures were applied: Response Surface Method (RSM), Taguchi procedure, and Artificial Neural Networks (ANN).

## 2. Optimization Window 

The phenomenon was studied both by simulation and experimentation using several different runner systems at various thermo-rheological material parameters and process operating conditions. Operating process parameters of the injection rate *V_inj_*, melt temperature *T_m_*, and mold temperature *T_M_*, as well as the geometry of the runner system were optimized, i.e., these were the optimized process parameters. The imbalance of the mold filling *I_m_* as well as the process parameters of injection pressure *P_inj_*, injection time *t_inj_*, and molding temperature *T_molding_* were the optimization criteria (Table 1).

Studies have been performed for an eight-cavity injection mold of “H-type” runner layout equipped with inserts of different geometry. A standard geometry GS have been used as well as three overturn geometries, G1 with one correction element, G2 with two correction elements, and G3 with circled element, these are depicted in Figure 2.

**Figure 3:**
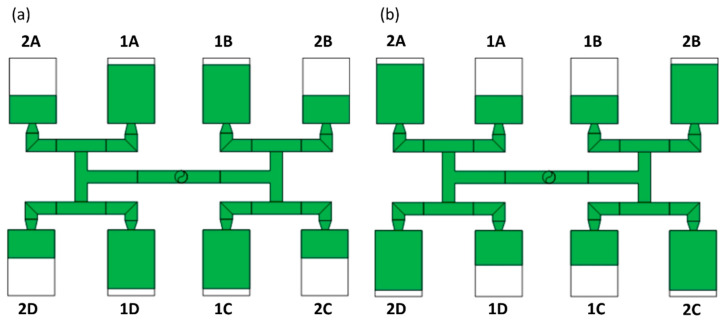
The concept of mass filling imbalance coefficient: (**a**) positive imbalance, *I_m_* > 0 (*m*_1_ > *m*_2_), faster filling of inner cavities; and (**b**) negative imbalance, *I_m_* < 0 (*m*_1_ < *m*_2_), faster filling of outer cavities.

The factor of mass filling imbalance *I_m_* [2] has been applied to evaluate the degree of imbalance which is defined as

(1) $$I_{m}=100\%\cdot \left(1-\frac{m_{2}}{m_{1}}\right)$$

where *I_m_* is the factor of mass filling imbalance, *m*_1_ is the mass of polymer from the inner cavities, and *m*_2_ is the mass of polymer from the outer cavities (Figure 3). 

The imbalance factor is positive when the inner cavities (1) fills faster *(I_m_ > 0)*, and it is negative when the outer cavities (2) filled faster *(I_m_* < 0). 

The optimization criteria determine the global objective function which was defined in the following way:

(2) $$F=\overset{4}{\underset{i=1}{\sum }}w_{i}f_{i}$$

where *F* is the global objective function, *i* is the number of optimization criteria, *w_i_* is the weight of the *i*-criterion, *f_i_* is the value of the normalized *i*-criterion.

The optimization criteria were specified as follows: *f*_1_ is the filling imbalance *I_m_*, *f*_2_ is the injection pressure *P_inj_*, *f*_3_ is the injection time *t_inj_*, and *f_4_* is the molding temperature *T_molding_*.

The values of the weights were as follows: *w*_1_ = 0.5 for the filling imbalance *I_m_*, *w*_2_ = 0.1 for the injection pressure *P_inj_*, *w*_3_ = 0.2 for the injection time *t_inj_*, and *w*_4_ = 0.2 for the molding temperature *T_molding_*. These are summarized in Table 2. 

The optimization criteria were normalized in the following way:

- when the optimization criterion is the minimum value of the output variable *y_i_*:
  (3) $$f_{i}=\frac{y_{max}-y_{i}}{y_{max}-y_{min}}$$
- when the optimization criterion is the maximum value of the output variable *y_i_*:
  (4) $$f_{i}=\frac{y_{i}-y_{min}}{y_{max}-y_{min}}$$

## 3. Optimization Procedures

Three optimization procedures were applied: Response Surface Methodology (RSM), Taguchi Method, and Brain Construction Algorithm (BCA) developed by STASA, which are shortly characterized below.

Response Surface Methodology (RSM) is a widely known and recognized statistical optimization method that explores the relationships between several input variables (independent variables) and one or more output variables (dependent or response variables) [26,27,28]. The main idea of RSM is to use a sequence of designed experiments to obtain an optimal response. However, a full factorial design is used in RSM, which means considering all combinations of parameter interactions, and it is time-consuming.

The RSM procedure consists of a full factorial experiment, approximation of the experimentation results (to do this, using a second-degree polynomial model is suggested), development of an extended set of data (to densify the response surface), and data normalization and selection based on the global objective function.

The Taguchi method [29,30] is based on the orthogonal array experiments, which provides much reduced variance for the study with an optimum setting of process control parameters. Thus, the integration of design of experiments (DOE) with parametric optimization of process to obtain desired results is achieved in the Taguchi method. Many experiments must be performed when the number of control factors is high. Taguchi methods use a special design of orthogonal arrays to study the entire factor space with only a small number of experiments.

The Taguchi method uses the signal-to-noise (S/N) ratio instead of the average value to convert the trial result data into a value for the characteristic in the optimum setting analysis. The S/N ratio reflects both the average and the variation of the quality characteristic. 

The standard S/N ratios generally used are as follows: *nominal is best* (*NB*), *lower the better* (*LB*), and *higher the better* (*HB*). The *larger (higher) the better* type characteristic (*HB*) is applied for problems where maximization of the quality characteristic of interest is sought. The *smaller (lower) the better* type characteristic (*LB*) is applied for problems where minimization of the characteristic is intended. The *nominal the best* characteristic (*NB*) is used for problems where one tries to minimize the mean squared error around a specific target value. 

Brain Construction Algorithm (BCA) is based on self-generating neural networks developed by STASA [31,32] that are linked with classical statistics. This has the flexibility and universality of neural models and the transparency of statistical theory. STASA has implemented a D-optimal design routine that tries to maximize the parameter space with a minimum of experiments. The result is an optimally adapted experimental design.

This novel optimization approach is based on the combination of mold filling numerical simulation and the BCA procedure implemented in the STASA QC software.

The procedure starts with the definition of optimization parameters and quality features (optimization criteria). Besides continuous measurable quality features such as dimensions or weight, also, attributive features that are evaluated in a more subjective way, e.g., surface glance, can be defined. 

In the next step, the software provides a design of experiment that is automatically created based on the parameters’ definitions. The experimental design provides a table with different parameter settings, which have to be set on the simulation consecutively. At every setting, the quality features of the simulated parts have to be measured. With this experimental design, the user receives guidance how to change the process setting systematically to receive the information about the interrelations between parameters and quality features required to generate the models. 

In practice, the number of experiments (different machine settings) in the experimental design is limited by cost factors. In general, no more than about 10 to 20 different machine settings are acceptable in practical applications. That is the reason the numerical calculation have been utilized. In spite of that, taking into account a full factorial design used in RSM, which means considering all combinations of parameter interactions, can only be used in exceptional cases. 

## 4. Optimization Results

### 4.1. Optimization by Response Surface Methodology (RSM) 

**Figure 4:**
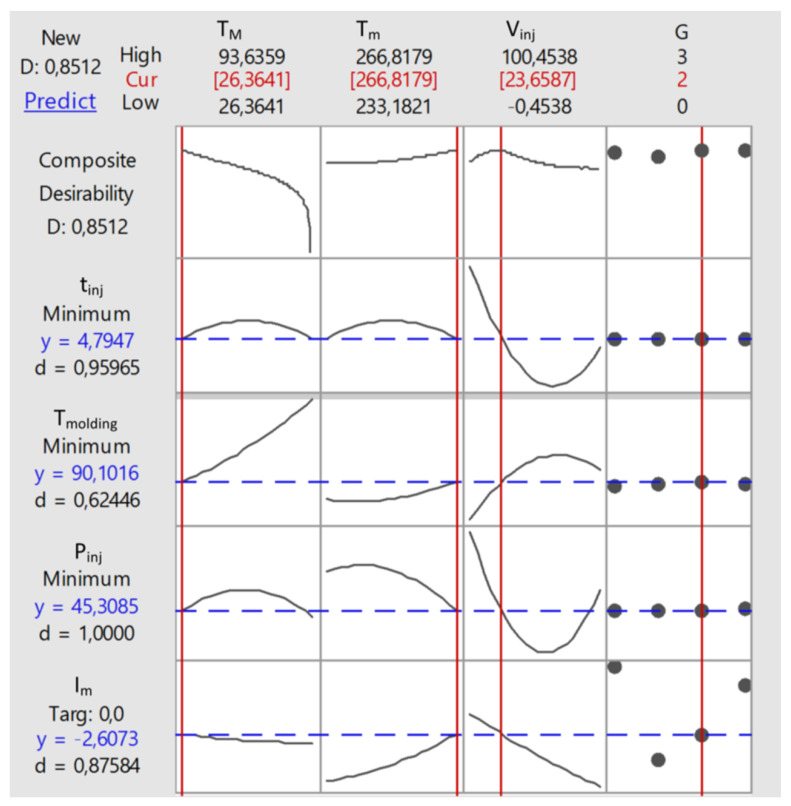
Graphical summary of RSM optimization results.

**Figure 5:**
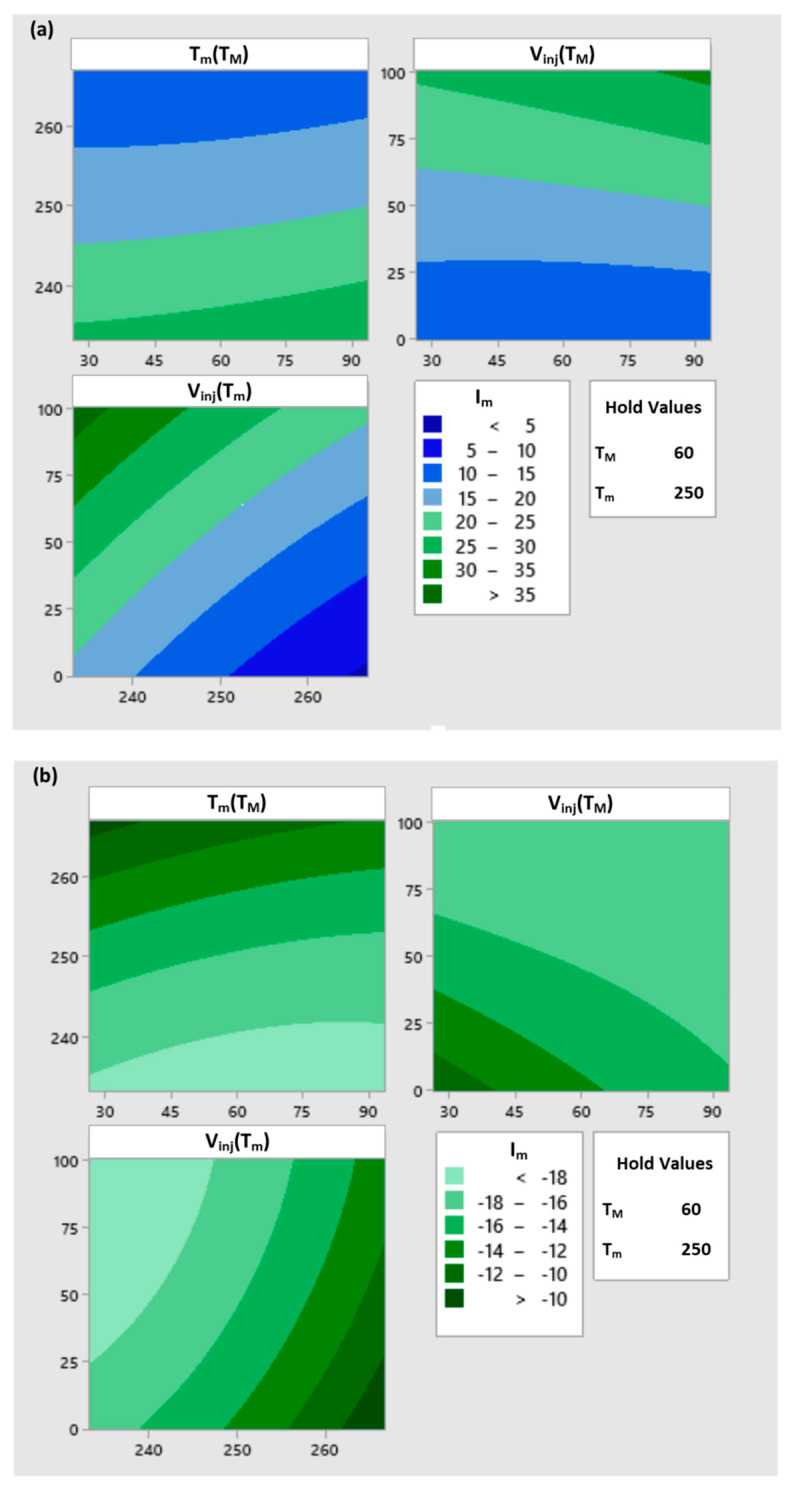

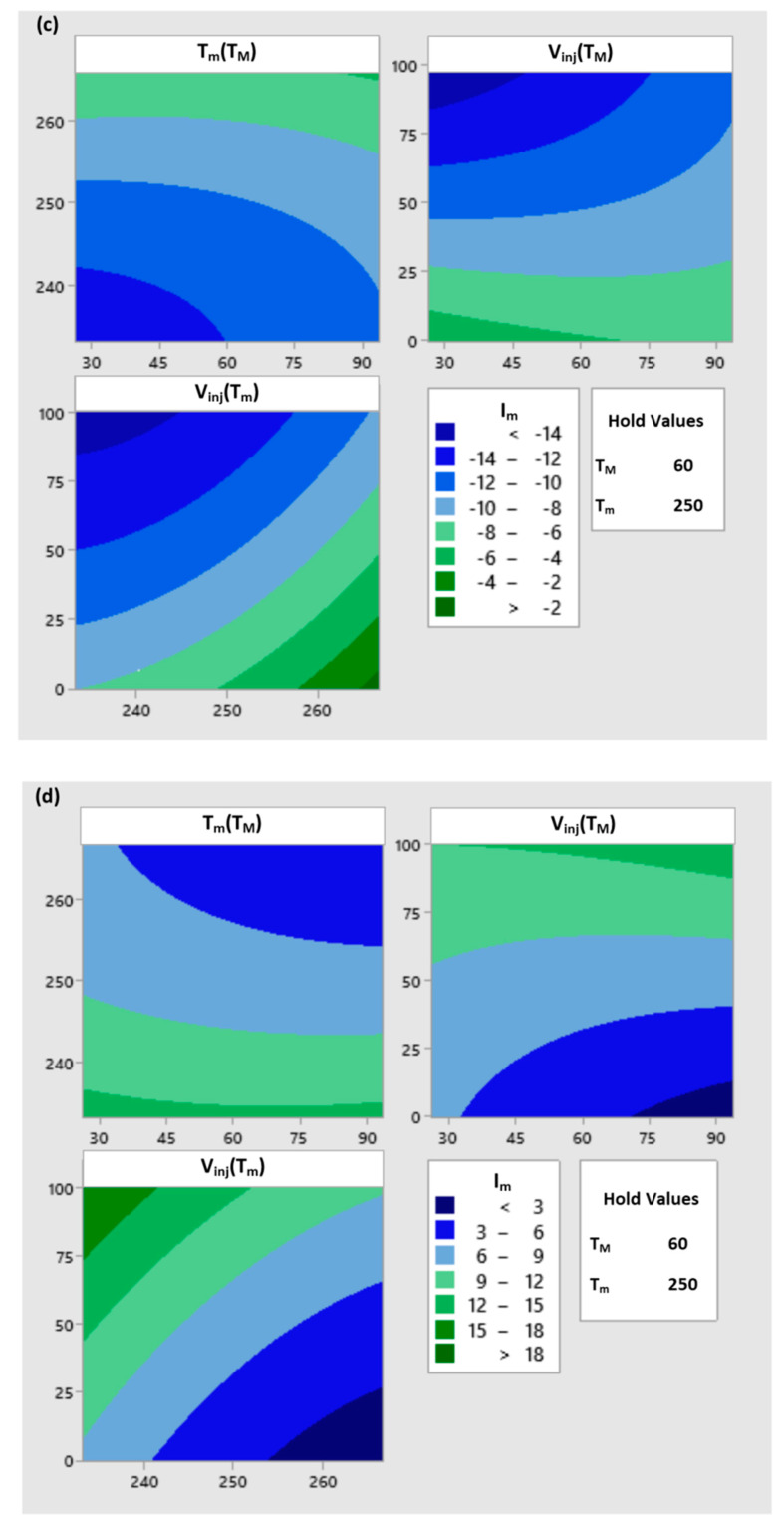
Effect of process parameters on the filling imbalance for various runner layouts: (**a**) standard GS, (**b**) one correction element G1, (**c**) two correction elements G2, (**d**) circled element G3.

**Figure 6:**
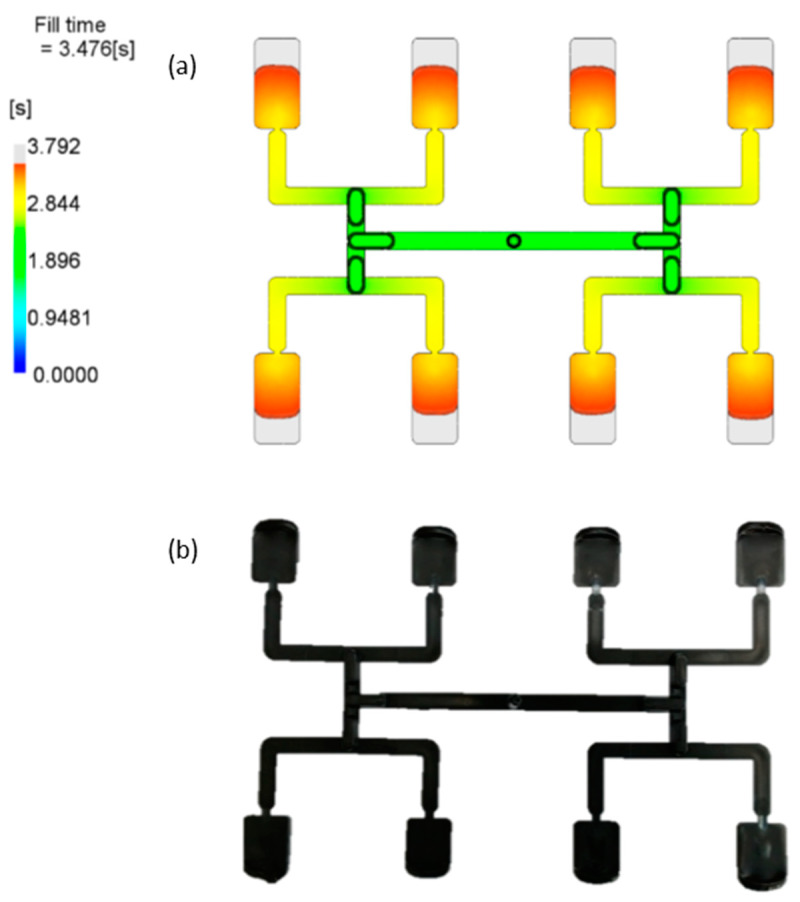
Results of simulation and experimentation for the optimum set of data (by Response Surface Methodology (RSM)): (**a**) simulation, (**b**) experiment.

The results of optimizations performed by RSM method are depicted in Figure 4 and Figure 5. Simulation and experimental results for optimum set of data are presented in Figure 6.

Figure 4 shows the optimum values of process parameters: the mold temperature *T_M_*, the melt temperature *T_m_*, the injection rate *V_inj_*, as well as the geometry of the runner system *G*, which are marked in red. The graphs show the optimum solution (red line) against the background of the desirability function (objective function). The optimum values of process parameters are listed in the conclusion table (Table 5). 

The composite (global) desirability dependence on each of the optimized parameters with the fixed (optimum) values of the other three parameters is presented in the first line. For example, the first graph from the left shows the dependence of the composite desirability *D* on the mold temperature *T_M_* at constant values of other parameters, i.e_.,_

(5) $${D=D\left(T_{M}\right)|}_{T_{m}=T_{m\;opt},\;V_{\;inj}=V_{\;inj\;opt},G=G_{opt}}$$

The individual desirability functions are shown in the next four lines with the values of these for the optimum set of data, i.e.,

$$d_{t{\;}_{inj\;}}\;\left(\;t_{inj}=4.7947\right)=0.95965$$

(6) $$d_{T_{molding}}\left(T_{molding}=90.1016\right)=0.6244$$

$$d_{P{\;}_{inj\;}}\left(P_{inj}=45.3085\right)=1.0000$$

$$d_{I_{m}}\left(I_{m}=-2.6073\right)=0.87584$$

Figure 5 presents the dependence (by regression) of the filling imbalance *I_m_* (output parameter) on each pair of two of the four input parameters (*T_M_*, *T_m_*, *V_inj_*, *G*) with fixed values (center of the variation range) of the other two parameters. For example, the graph in the upper left corner shows the dependence of the filling imbalance *I_m_* on the mold temperature *T_M_* and the melt temperature *T_m_* at the constant values *V_inj_* = 50 mm/s and *G* = 0.

(7) $${I_{m}=I_{m}\left(T_{M},T_{m}\right)|}_{\;V_{\;inj}=50\;\mathrm{mm}/s,\;\;\;G=0}$$

Figure 6 presents simulation and experimental results for optimum set of data. It is clearly seen that the imbalance is relatively low, and equal to about *I_m_* = −9%, which means the outer cavities are filled faster.

### 4.2. Optimization by Taguchi Method

The Taguchi method implemented into the Moldex3D software has some limitations. The optimization window is defined by the limited number of optimized parameters. For that reason, we assumed (from the available data) the following parameters (control factors, optimized parameters): the mold temperature *T_M_*, the melt temperature *T_m_*, the filling time *t_fill_*, and the mesh size *M*. In this approach, the filling time *t_fill_* corresponds to the injection rate *V_inj_*, and the mesh size *M* corresponds to the runner geometry *G*. The range of these data is presented in Table 3. 

The optimization criteria (quality factors) are also limited, and were selected as follows: the density distribution *D_distrib_*, the average temperature distribution *T_av_*, and the sprue injection value *P_sprue_*. These factors correspond to the filling imbalance *I_m_*, the molding temperature *T_molding_*, and the injection pressure *P_inj_*. The characteristics of these and their weighting is presented in Table 4.

**Figure 7:**
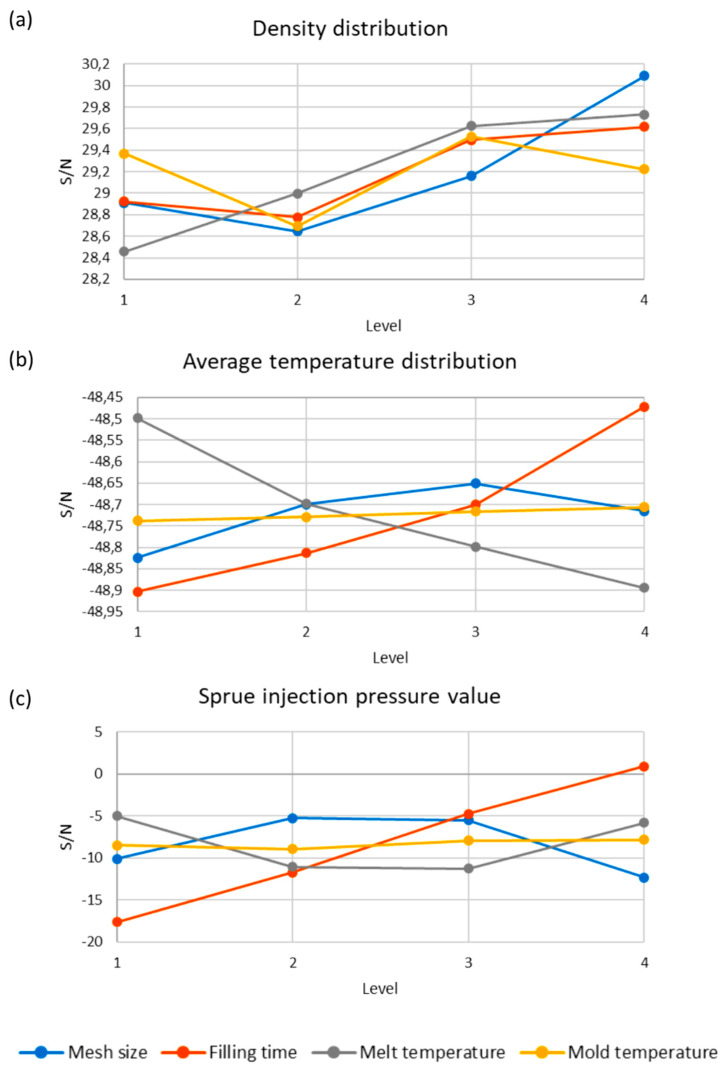
Signal-to-noise (S/N) response curves for various control factors: (**a**) density distribution, (**b**) average temperature distribution, (**c**) sprue injection pressure.

The optimal setting is the parameters combination that has the highest S/N ratio. For example, the filling time has the highest S/N ratio for the set of control factors (optimized parameters) defined by Level 4 (Figure 7).

In the paper, *smaller the better* characteristic has been used for the average temperature distribution *T_av_* and the sprue injection pressure *P_sprue_*, and *nominal the best* characteristic for the density distribution *D_distrib_* (Table 4).

**Figure 8:**
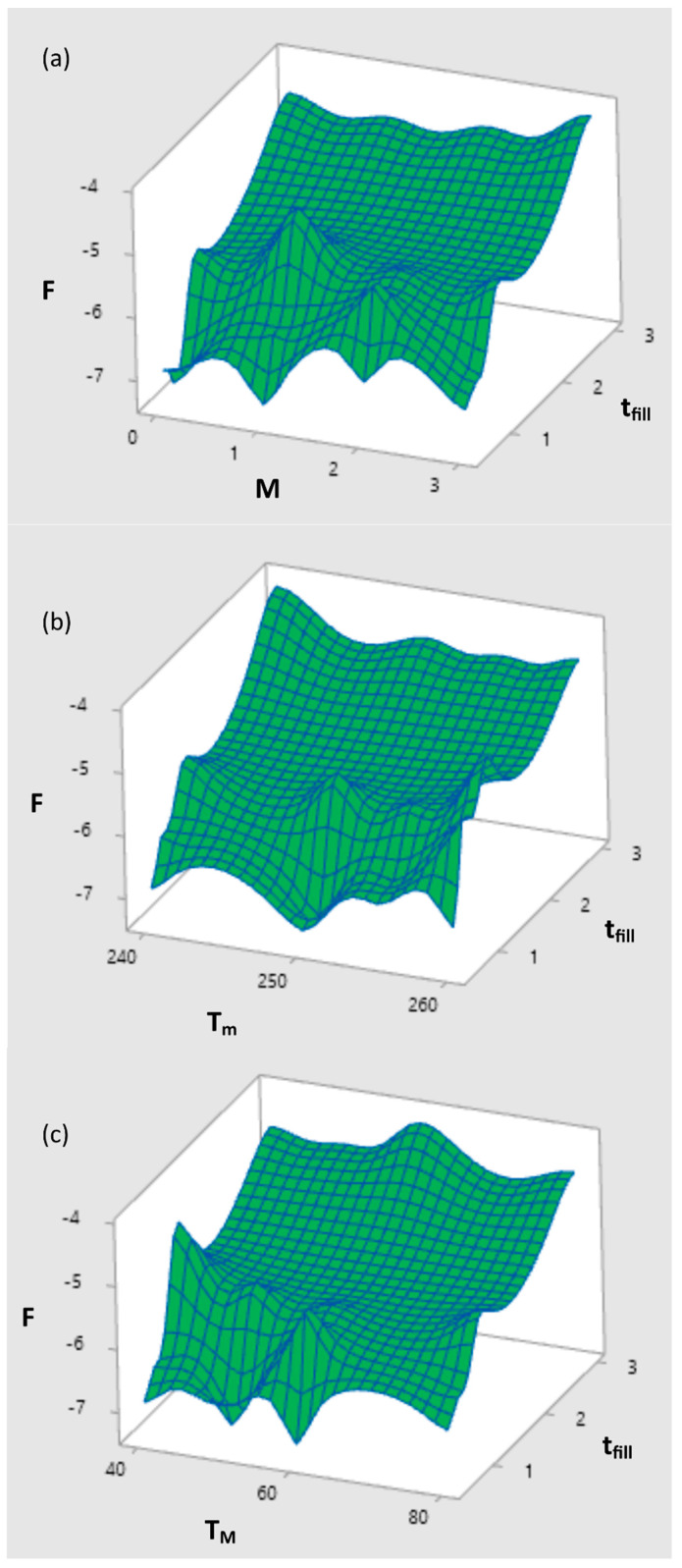
Response surface plot *F* (global objective function) vs. *t_fill_* and (**a**) vs. *M* for *T_M_ = T_M opt_, T_m_ = T_m opt_*, (**b**) vs. *T_m_* for *T_M_ = T_M opt_, G = G_opt_*, (**c**) vs. *T_M_* for *T_m_ = T_m opt_*, *G = G_opt_*.

**Figure 9:**
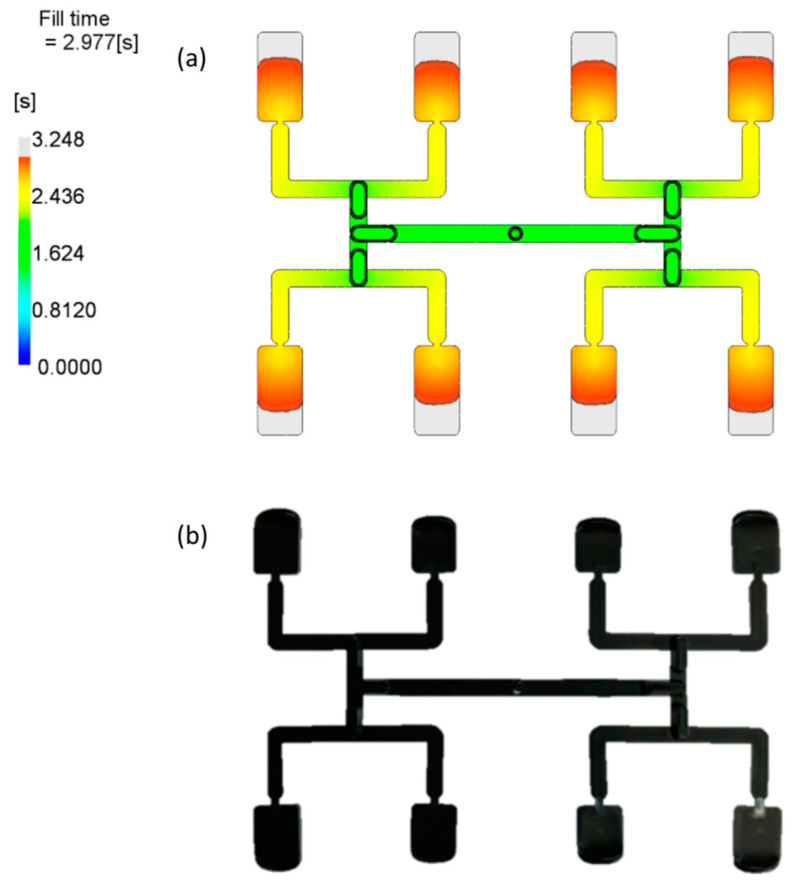
Results of simulation and experimentation for the optimum set of data (by Taguchi): (**a**) simulation, (**b**) experiment.

The results of optimizations performed by Taguchi method are depicted in Figure 7 and Figure 8. Simulation and experimental results for optimum set of data are presented in Figure 9.

Figure 9 presents simulation and experimental results for optimum set of data. It is clearly seen that the imbalance is relatively low, and equal to about *I_m_* = −14%, which means the outer cavities are filled faster.

### 4.3. Optimization by Neural Network STASA

**Figure 10:**
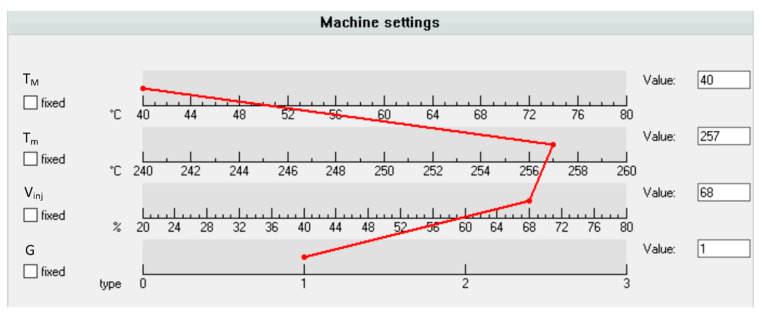
Optimized parameters by STASA QC: *T_M_*—mold temperature, *T_m_*—melt temperature, *V_inj_*—injection rate, *G*—geometry.

**Figure 11:**
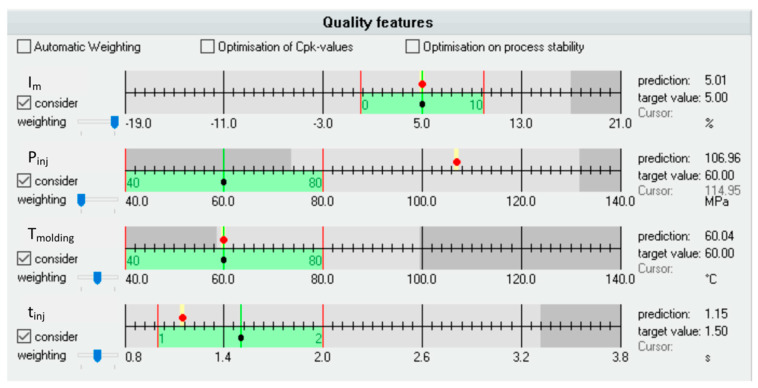
Optimization criteria for optimal setting with respect to predefined range: (**a**) *I_m_*—imbalance (**b**) *P_inj_*—injection pressure, (**c**) *T_molding_*—molding temperature, (**d**) *t_inj_*—injection time.

**Figure 12:**
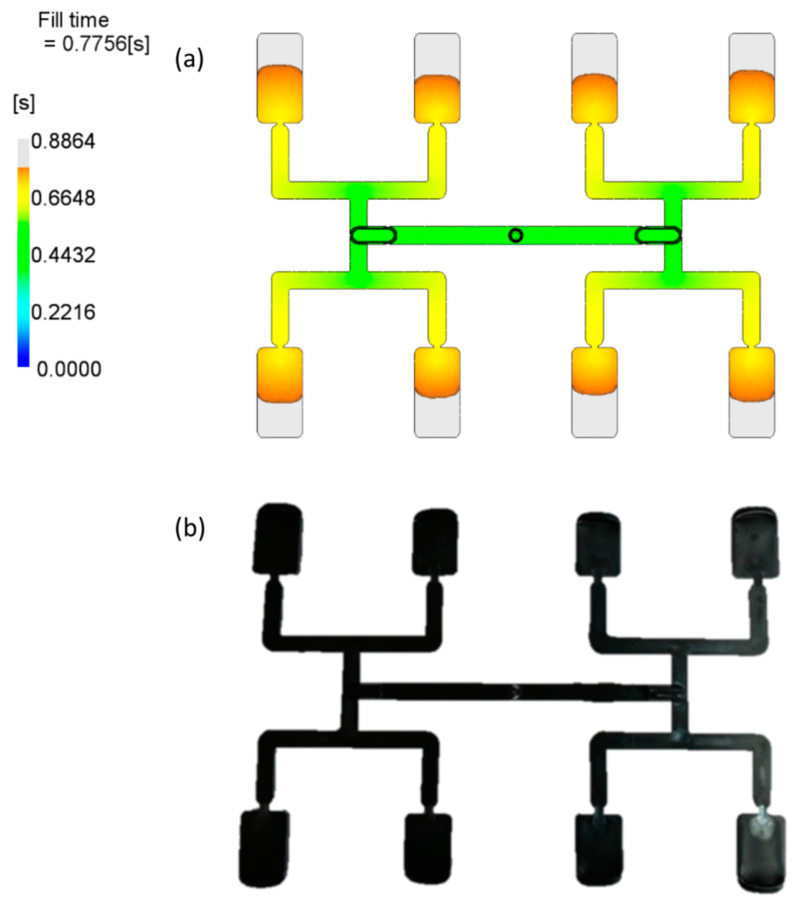
Results of simulation and experimentation for the optimum set of data (by STASA QC): (**a**) simulation, (**b**) experiment.

The results of optimizations performed by STASA software are depicted in Figure 10 and Figure 11. Simulation and experimental results for optimum set of data are presented in Figure 12.

Figure 10 shows the optimum values of process parameters: the mold temperature *T_M_*, the melt temperature *T_m_*, the injection rate *V_inj_*, as well as the geometry of the runner system *G*, which are equal to: *T_M_* = 40 °C, *T_m_* = 257 °C, *V_inj_* = 68 mm/s, *G* = 1, respectively. The optimum is described by the red line that connects the optimal parameter values.

The optimum values of process parameters are listed in the conclusion table (Table 5).

The values of optimization criteria for optimal setting with respect to the predefined range of searching are presented in Figure 11. The red dots indicate the values of the criteria, the black dots represent the predefined target values, and the green areas represent the range of searching.

Figure 12 presents simulation and experimental results for optimum set of data. It is clearly seen that the imbalance is relatively low, and equal to about I_m_ = −9%, which means the outer cavities are filled faster.

## 5. Discussion 

The optimization results are summarized in Table 5. The optimal values of process parameters (*V_inj_*, *T_m_, T_M_, G*) and the corresponding values of optimization criteria (*I_m_*, *P_inj_*, *t_inj_*, *T_molding_*), as well as the values of global objective function *F* are given. The simulation results were obtained using Moldflow software, the prediction results were received from optimization software by calculation on the base of their own models built on the simulation data. The experimental results were obtained by performing the experiment at the optimal process parameters. 

The highest value of the global objective function has been predicted for the optimal process parameters indicated by BCA method (STASA QC), and also for these optimal parameters, the highest value of the experimental global objective function has been obtained. So, it seems to be justified that the Artificial Neural Networks approach is the most efficient optimization procedure, and the Brain Construction Algorithm (BCA) might be proposed for problem solving of imbalance phenomena.

**Figure 13:**
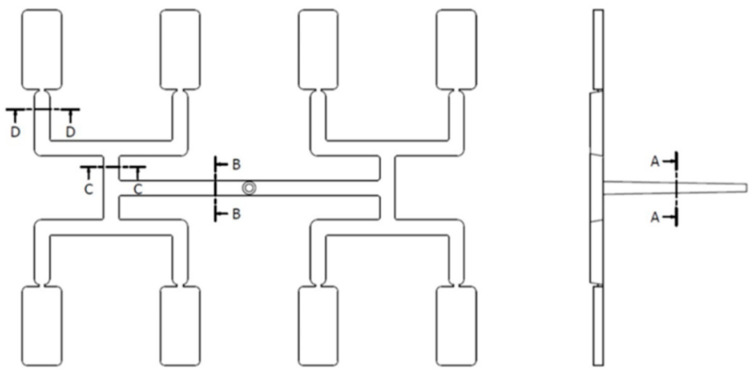
Specific cross-sections of the runner system.

**Figure 14:**
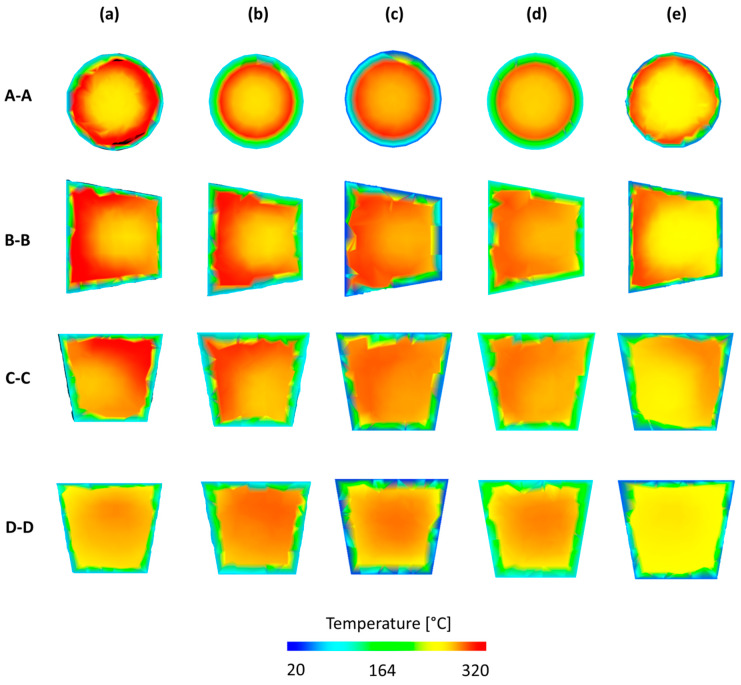
Temperature plots in specific cross-sections of the runner system obtained from simulations performed on different data, and using different optimization procedures: (**a**) maximum positive imbalance (*GS*), (**b**) maximum negative imbalance (*G*2), (**c**) optimum RSM (*G*2), (**d**) optimum Taguchi (*G*2), (**e**) optimum STASA *(G)*1.

However, it is important to note that all the optimization procedures substantially improved the filling balance, which is also confirmed by simulations depicted in Figure 13 and Figure 14. 

Temperature plots in specific cross-sections of the standard runner system (Figure 13) obtained from simulations performed on different data, and using different optimization procedures are depicted in Figure 14. A maximum positive imbalance and maximum negative imbalance, as well as optimum data for RSM, Taguchi, and STASA are shown. It is clearly seen that for the standard geometry *GS,* the polymer melt stream rotates to the right (cross-section C–C, Figure 14a), which results in a positive imbalance. While, for the two overturn geometry *G2* the polymer melt stream rotates to the left (cross-section C–C, Figure 14b), which results in a negative imbalance. In the optimized cases, there is no substantial melt stream rotation that leads to much more balanced flow and cavity filling. It is worth noting that rotation is observed in all cases; however, the degree of rotation varies. 

**Figure 15:**
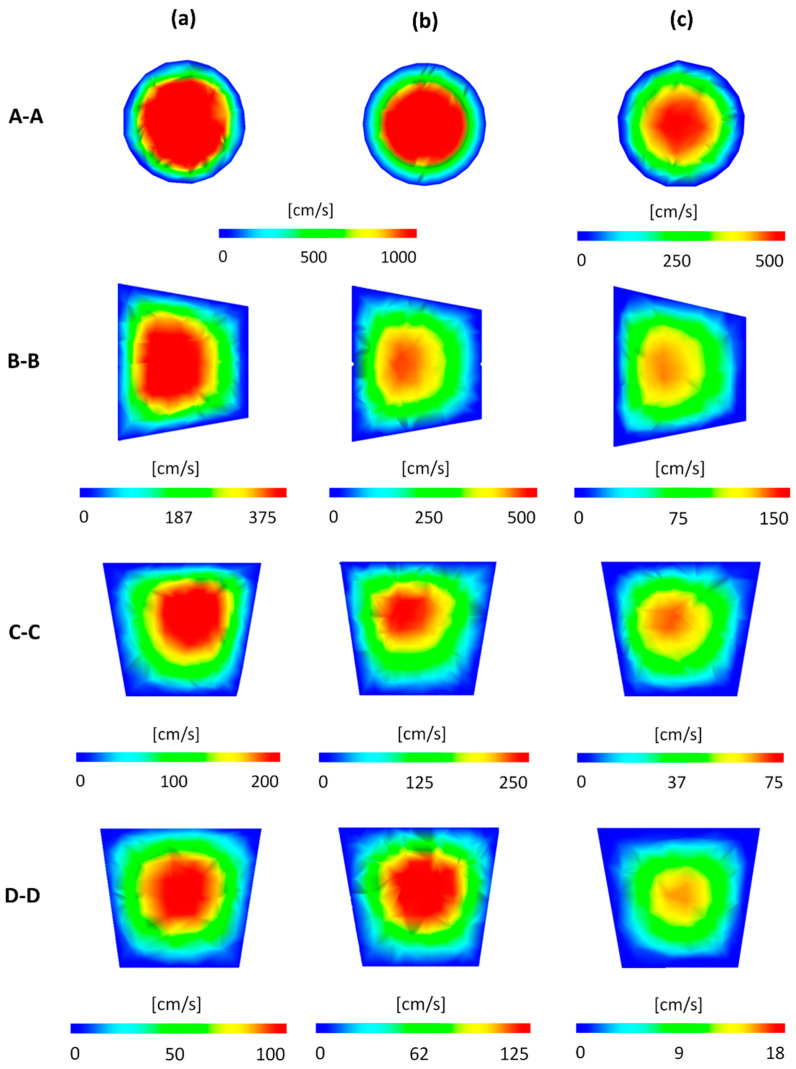
Velocity plots in specific cross-sections of the runner system obtained from simulations performed on different data, and using STASA optimization procedures: (**a**) maximum positive imbalance (*GS*), (**b**) maximum negative imbalance (*G*2), (**c**) optimum STASA (*G*1). The optimized data obtained by simulation have been compared with the experimental values, and the relative errors have been listed in Figure 16. This comparison is limited to the RSM method and BCM procedure since the Taguchi method has some parameter limitations (see Section 4.2).

Velocity plots in specific cross-sections of the runner system (Figure 13) obtained from simulations performed on the data providing a maximum positive imbalance and maximum negative imbalance as well as optimum data for STASA system are depicted in Figure 15. The most balanced flow is clearly seen for the data indicated by STASA (Figure 15c).

The comparison is related to the simulation validation, not to the optimization. An accuracy of simulation is quite good, especially in the case of BCM procedure. In this case, the optimized value of injection rate *V_inj_* was very high. It results from our previous studies that discrepancies between simulation and experimentation data substantially increase when injection rate *V_inj_* increases [1].

## 6. Conclusions

Three optimization techniques were used to study and optimize the filling imbalance in geometrically balanced injection molds: Response Surface Methodology (RSM), Taguchi method, and Artificial Neural Networks (ANN). The process parameters of injection rate *V_inj_*, melt temperature *T_m_*, and mold temperature *T_M_*, as well as the geometry of the runner system G were optimized. The mold filling imbalance *I_m_* as well as the process parameters of injection pressure *P_inj_*, injection time *t_inj_*, and molding temperature *T_molding_* were the optimization criteria.

It can be concluded that using the optimization procedures improves the filling imbalance; however, the Artificial Neural Networks using the Brain Construction Algorithm (BCA) seems to be the most efficient optimization procedure. 

It can be also noted that a comprehensive approach to modeling of injection molding may be useful for simulation of the flow in injection molds and for the prediction of the filling imbalance. A global injection molding model might be considered for simulation of the polymer melt flow in the plasticating unit of the injection molding machine as well as in the mold. Resulting parameters of the plasticating unit simulations would be input data for the mold flow simulations. Some of the injection molding studies with this respect were recently discussed by the authors [1,2].

## Figures and Tables

**Figure 16:**
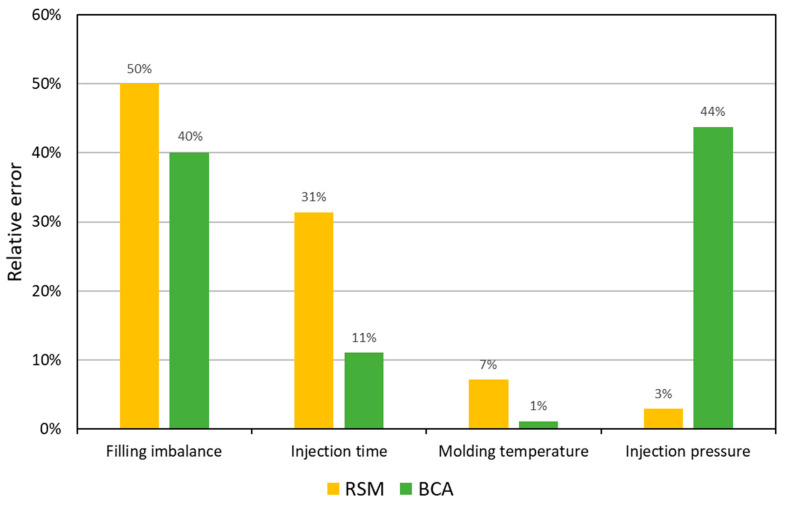
Relative error of experimental validation of simulations performed on the optimized data: filling imbalance *I_m_* (*w*_1_ = 0.5), injection time *t_inj_* (*w*_4_ = 0.2), molding temperature *T_molding_* (*w*_3_ = 0.2), injection pressure *P_inj_* (*w*_2_ = 0.1).

**Table 1:** Range of optimized process parameters.

Name	Symbol	Unit	Type	Min Value	Max Value
Melt temperature	*T_m_*	°C	continuous	240	260
Mold temperature	*T_M_*	°C	continuous	40	80
Injection rate	*V_inj_*	mm/s	continuous	20	80
Runner geometry	*G*	N/A	discrete	0	3

**Table 2:** Optimization criteria.

Name	Symbol	Symbol of Normalized Criterion	Weight of the Criterion	Weight Value
Filling imbalance	*I_m_*	*f*_1_	*w*_1_	0.5
Injection pressure	*P_inj_*	*f*_2_	*w*_2_	0.1
Injection time	*t_inj_*	*f*_3_	*w*_3_	0.2
Molding temperature	*T_molding_*	*f*_4_	*w*_4_	0.2

**Table 3:** Specified range of control factors (optimized process parameters) for Taguchi method.

Control Factor	Level 1	Level 2	Level 3	Level 4
Mesh size *M*	*GS*	*G*1	*G*2	*G*3
Filling time, s *t_fill_*	0.5	0.75	1.2	3
Melt temperaturę, °C *T_m_*	240	250	255	260
Mold temperaturę, °C *T_M_*	40	50	60	80

**Table 4:** Definition of quality factors (optimization criteria) for Taguchi method.

Quality Factor	Characteristic	Weighting
Average temperature distribution *T_av_*	*Smaller the better*	0.4
Density distribution *D_distrib_*	*Nominal the best*	0.5
Sprue injection pressure *P_sprue_*	*Smaller the better*	0.1

**Table 5:** Results of optimization.

		Optimal Parameters				Values of Optimization Criteria				Objective Function
		*V_inj_* [mm/s]	*T_m_* [°C]	*T_M_* [°C]	*G*	*I_m_* [%]	*T_molding_* [°C]	*t_inj_* [s]	*P_inj_* [MPa]	*F*
Taguchi	Simulation	20	260	60	2	−4	94.0	3.01	48.3	0.80205
	Experiment					−14	96.6	3.0	45.4	0.632866
RSM	Prediction	24	266	26	2	−2.6	92.0	4.9	46.0	0.725025
	Simulation					−6	90.8	3.5	44.3	0.760807
	Experiment					−9	97.3	2.4	43,0	0.755103
BCA	Prediction	68	257	40	1	5	60.0	1.2	106.9	0.886497
	Simulation					−15	61.3	0.9	102.1	0.74406
	Experiment					−9	62.0	0.8	57.4	0.951511
